# Emerging roles of adhesion G protein-coupled receptors

**DOI:** 10.1042/BST20201144

**Published:** 2021-07-20

**Authors:** Matthew Rosa, Timothy Noel, Matthew Harris, Graham Ladds

**Affiliations:** Department of Pharmacology, University of Cambridge, Tennis Court Road, Cambridge CB2 1PD, U.K.

**Keywords:** adhesion receptors, agonists, G-protein-coupled receptors, G-proteins, signal transduction

## Abstract

Adhesion G protein-coupled receptors (aGPCRs) form a sub-group within the GPCR superfamily. Their distinctive structure contains an abnormally large N-terminal, extracellular region with a GPCR autoproteolysis-inducing (GAIN) domain. In most aGPCRs, the GAIN domain constitutively cleaves the receptor into two fragments. This process is often required for aGPCR signalling. Over the last two decades, much research has focussed on aGPCR-ligand interactions, in an attempt to deorphanize the family. Most ligands have been found to bind to regions N-terminal to the GAIN domain. These receptors may bind a variety of ligands, ranging across membrane-bound proteins and extracellular matrix components. Recent advancements have revealed a conserved method of aGPCR activation involving a tethered ligand within the GAIN domain. Evidence for this comes from increased activity in receptor mutants exposing the tethered ligand. As a result, G protein-coupling partners of aGPCRs have been more extensively characterised, making use of their tethered ligand to create constitutively active mutants. This has led to demonstrations of aGPCR function in, for example, neurodevelopment and tumour growth. However, questions remain around the ligands that may bind many aGPCRs, how this binding is translated into changes in the GAIN domain, and the exact mechanism of aGPCR activation following GAIN domain conformational changes. This review aims to examine the current knowledge around aGPCR activation, including ligand binding sites, the mechanism of GAIN domain-mediated receptor activation and how aGPCR transmembrane domains may relate to activation. Other aspects of aGPCR signalling will be touched upon, such as downstream effectors and physiological roles.

## Introduction

G protein-coupled receptors (GPCRs) are currently the most successfully targeted superfamily of receptors in modern medicine [1]. GPCRs are classified into five main families; Glutamate, Rhodopsin, Frizzled/Taste, Secretin and importantly for this review, Adhesion [2]. They are responsible for a large variety of cellular responses with a diverse selection of stimuli, resulting in a complex network of interactions between the ligands, the receptors and the signalling cascade. Whilst GPCRs in general are the most targeted receptor superfamily, historically very little pharmaceutical research has been conducted on adhesion GPCRs (aGPCRs). Despite their importance in adhesion, cell migration, paracrine signalling and numerous disease implications [3], aGPCR research has been hampered by the orphan status of many receptors. Nonetheless, aGPCRs provide an intriguing potential alternative drug target compared with many other families, in particular, within oncology and fertility. Several recent reviews have highlighted the emerging role of these receptors in therapeutics therefore it is not the aim of this review to reiterate these points [4,5]. Instead, here we aim to discuss the current understanding of known aGPCR ligands, their activation, structure and function.

## aGPCR nomenclature has drastically changed since their discovery

What we know today as aGPCRs were first characterised in leukocytes in the 1980s. They were identified as the glycoproteins targeted by the mouse monoclonal antibody for F4/80, the mouse equivalent of the human GPCR EMR1 [6]. F4/80, EMR1 and CD97 were the first members of the GPCR subfamily originally known as EGF-TM7; named for the appearance of F4/80 as a chimera of 7-transmembrane receptors and epidermal growth factor (EGF) [7,8].

A misleading alternative name for this family was LNB-TM7, denoting its long N-terminal region, but also a close association with Class B1 GPCRs [9]. Early reviews often listed these GPCRs as a subfamily of Class B GPCRs, due to their sequence similarity in the 7 transmembrane helix domain (7TM) [10,11]. However, analysis of the entire GPCR superfamily revealed distinctions between this family and Class B1, in particular in the extracellular domain (ECD). This new family, with 24 members at the time, was named ‘adhesion’, for their apparent role in cell adhesion due to the mucin-like stalks in their N-terminal region [12,13]. Subsequently, all 33 human family members were divided further into nine clusters (Table 1), with each having a relatively high sequence similarity that the family lacks as a whole [14]. The International Union of Basic and Clinical Pharmacology defined the family fully in 2015 [15].

**Table 1:** A summary of known endogenous ligands, receptor activation mechanisms, G protein couplings and domains contained in the NTF, N-terminal to the GAIN domain, of every human aGPCR

Cluster	aGPCR	Determined ligand(s)	Activation mechanism^1^	Established G proteins couplings	N-terminal domain(s)	Source
I	ADGRL1 (Latrophilin-1)	Teneurin-2, FLRT1, FLRT3, neurexin-1α, -1β, -2β	Tethered agonist (A)/constitutively active mutants (C)	G_s_, G_i_	Lectin, olfactomedin, STP, HomR	[77–86]
	ADGRL2 (Latrophilin-2)	Teneurin-2, FLRT3	Unknown	Unknown		
	ADGRL3 (Latrophilin-3)	Teneurin-3, FLRT1, FLRT3, Unc5D	Tethered agonist (A)	G_12_, G_13_		
	ADGRL4 (ELTD1)	-	Unknown but not tethered agonist or constitutive activity	Unknown	Lectin, EGF-Like, 2× Ca^2+^-binding EGF	
II	ADGRE1 (EMR1)	-	Unknown	Unknown	EGF-Like, 5× Ca^2+^-binding EGF	[16,40,49,53,76,87–93]
	ADGRE2 (EMR2)	Chondroitin sulfate B, FHR1	Unknown/constitutive activity (C)	G_16_	EGF-Like, 4× Ca^2+^-binding EGF	
	ADGRE3 (EMR3)	-	Unknown	Unknown	EGF-Like, 1× Ca^2+^-binding EGF	
	ADGRE4 (EMR4)	-	Unknown/not expressed at cell surface	Unknown		
	ADGRE5 (CD97)	CD55, chondroitin sulfate B, integrins α_5_β_1_ and α_v_β_3_, CD90	Tethered agonist (A)/constitutive activity (C)	G_12_, G_13_, G_14_, G_z_	EGF-Like, 4× Ca^2+^-binding EGF, RGD motif	
III	ADGRA1 (GPR123)	-	No GAIN domain present therefore not tethered agonist	Unknown	-	[14,22,94–97]
	ADGRA2 (GPR124)	Integrin α_v_β_3_, glycosaminoglycans, syndecan-1,2	Unknown	Unknown	LRR, IG, RGD motif, HomR	
	ADGRA3 (GPR125)	-	Constitutive activity (C)	Unknown	LRR, IG, HomR	
IV	ADGRC1 (CELSR1)	-	Unknown but not tethered agonist	Unknown	EC, 5× Ca^2+^-binding EGF, 2× LamG, EGF-Lam, HomR	[39,98–101]
	ADGRC2 (CELSR2)	-	Tethered agonist (A)/constitutive activity (C)	Potentially G_q_		
	ADGRC3 (CELSR3)	Dystroglycan	Tethered agonist (A)	Potentially G_q_	EC, 5× Ca^2+^-binding EGF, 2× LamG, 2× EGF-Lam, HomR	
V	ADGRD1 (GPR133)	Plxdc2	Tethered agonist (A)/constitutive activity (C)	G_s_	-	[14,102–104]
	ADGRD2 (GPR144)	-	Unknown	Unknown	PTX	
VI	ADGRF1 (GPR110)	Synaptamide	Soluble ligand allosteric binding (B)	G_q_, G_s_	SEA	[14,23,96,105–112]
	ADGRF2 (GPR111)	-	Unknown but not tethered agonist	Unknown	-	
	ADGRF3 (GPR113)	-	Unknown	Unknown	HomR, EGF	
	ADGRF4 (GPR115)	-	Unknown but not tethered agonist	Unknown	-	
	ADGRF5 (GPR116)	Surfactant protein D	Tethered agonist (A)	G_q_, G_11_	SEA, 2× IG	
VII	ADGRB1 (BAI1)	Phosphatidylserine, integrin α_v_β_5_, lipopolysaccharide, RTN4R, CD36	Tethered agonist (A)	G_12_, G_13_	RGD motif, 5× TSR, HomR	[14,31,95,113–123]
	ADGRB2 (BAI2)	Glutaminase interacting protein	Tethered agonist (A)	G_z_, G_i_	4× TSR, HomR	
	ADGRB3 (BAI3)	C1ql-1,4	Unknown	Unknown	CUB, 4× TSR, HomR	
VIII	ADGRG1 (GPR56)	Collagen III, tissue transglutaminase 2, laminin	Tethered agonist (A)	G_i_, G_q_	-	[46,54,62,67,96,106,124–134,134–138]
		Progastrin				
	ADGRG2 (GPR64, HE6)	-	Constitutive activity (C)	G_q_, G_11_	-	
	ADGRG3 (GPR97)	Cortisol*	Soluble ligand (TM binding) (B)/constitutive activity (C)	G_s_, G_i_, G_o_	-	
	ADGRG4 (GPR112)	-	Unknown	Unknown	PTX, RGD motif	
	ADGRG5 (GPR114)	-	Tethered agonist (A)/constitutive activity (C)	G_s_	-	
	ADGRG6 (GPR126, VIGR, DREG)	Collagen IV, laminin-211	Tethered agonist (A)	G_s_, G_i_, G_o_	CUB, PTX, SEA, HomR	
		Cellular prion protein				
	ADGRG7 (GPR128)	-	Unknown	Unknown	-	
IX	ADGRV1 (GPR98, VLGR1)	-	Tethered agonist (A)	Unknown	35× CB, PTX, EAR	[42,109]

1 Letters in brackets denote the panel from Figure 3 that illustrates the activation mechanism used by each aGPCR;

CB: Calx-beta motif; CUB: Complement C1r/C1s, Uegf, Bmp1; EAR: Epilepsy-associated repeat; EC: Extracellular cadherin domains (9 cadherin repeats); EGF: Epidermal growth factor; FHR: Factor H-related protein; FLRT: fibronectin leucine-rich transmembrane protein; HomR: Hormone receptor; IG: Immunoglobulin; LamG: laminin-G like domain; LRR: Leucine-rich repeat domain; PTX: Pentraxin; SEA: Sperm protein, enterokinase and agrin; STP: Ser/Thr/Pro-rich domain; TSR: Thrombospondin type 1 repeat. Ligands shown in red are soluble and act while not anchored to a cell or extracellular matrix. List of ligands adapted from Vizurraga et al. [22].

## Endogenous ligands of aGPCR

**Figure 1.**
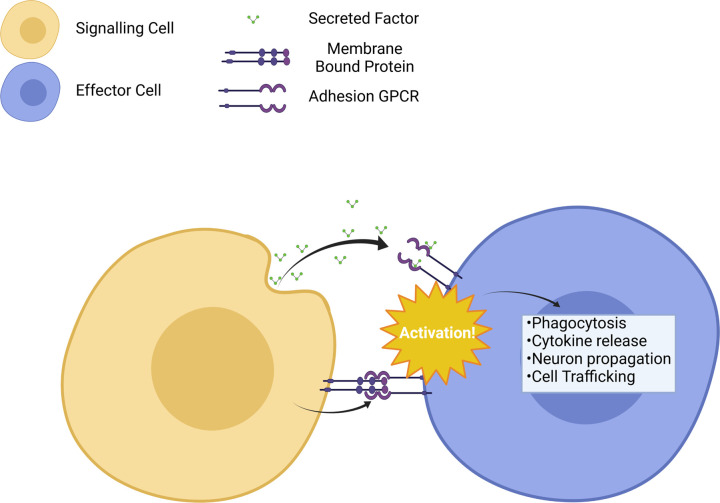
Types of signalling between cells using aGPCRs. aGPCRs are mainly utilised in paracrine or autocrine signalling via either secreted factors (top) or membrane-bound proteins and proteoglycans on adjacent cells (bottom). Activation through either of these two methods can lead to a cellular response. Created with Biorender.

Due to their role in cell-to-cell adhesion, it is unsurprising that several ‘anchor points’ such as receptors and proteins typically found in the plasma membrane can potentially activate aGPCRs [16]. A schematic representing this endogenous paracrine activation is portrayed in Figure 1. Phospholipids, such as phosphatidylserine (PS), are an integral part of the plasma membrane, involved in numerous cell signalling events, whilst exofacial PS is a key marker of apoptotic events [17]. PS can activate the brain specific angiogenesis inhibitor 1 (BAI1, ADGRB1) found on microglial cell surfaces and cause engulfment of the presenting cell. Further membrane-bound proteins associated with aGPCRs include the lysophosphatidic acid receptor (LPA1) present on the vast majority of mammalian tissues which can bind to CD97 (ADGRE5) found on lymphoid and myelinoid cells increasing the signalling of LPA1 [18]. Activation promotes adhesion and migration to sites of inflammation.

Aside from membrane bound proteins, secreted factors are also known to activate aGPCRs (Figure 1) [19]. These include secreted peptides and proteoglycans typically found in the extracellular fluid or the tissue stroma around the body. This is where the original classification of the aGPCRs, and their most closely related family, the Class B1 GPCRs showed their similarity with both classes activated through hydrophilic peptides. Whilst this is the case for some aGPCRs, many other non-peptide ligands have already been documented for aGPCRs (Table 1) [20]. Soluble aGPCR ligands are typically glycosaminoglycans, such as chondroitin sulfate found in lung and pancreatic tissue [21]. Other soluble ligands include proteins such as glutaminase interacting protein (GIP) as well as small molecules such as synaptamide, an endocannabinoid-like derivative [22,23]. This varied subset of ligands suggests a multifaceted role for these receptors outside of simply cell-to-cell adhesion and paracrine signalling. Despite their broad distribution and novel screening techniques, 17 of the 33 known aGPCRs are still without known endogenous ligands (Table 1), membrane-bound or unbound [24], with significant efforts focused on deorphanisation.

## aGPCR structure is separated into two fragments, each with conserved and variable regions

**Figure 2.**
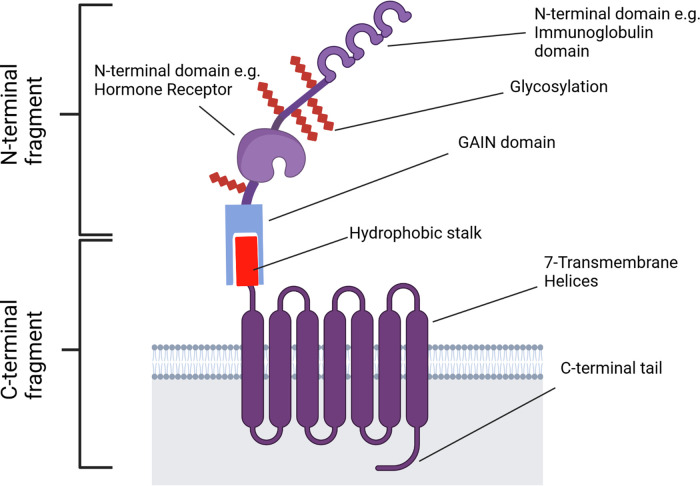
Example aGPCR structure. The GPS, dividing the N- and C-terminal fragments, lies between the hydrophobic stalk and GAIN domain. Created with Biorender.

aGPCRs are made up of two major components: N- and C-terminal fragments (NTF and CTF, respectively). The NTF encompasses most of the protein's ECD, comprising the GPCR autoproteolysis-inducing (GAIN) domain and a large, heavily glycosylated N-terminal region that varies in structure between each individual aGPCR and aGPCR sub-group. The CTF is C-terminal to the GAIN domain's GPCR proteolysis site (GPS), comprising the 7TM domain and an intracellular C-terminal tail (Figure 2).

## aGPCR activation mechanisms suggest stalk and lever function

Due to the initial lack of endogenous ligands and modern techniques, initial exploration into the signalling of aGPCRs was slow. Despite having initially been placed into the Class B1 GPCR family, the large ECD lent itself to the ligand-binding site theory, like Class C GPCRs [25]. However, removal of part of the ECD increased receptor activity, contrary to the initial prediction of it decreasing due to the loss of the orthosteric site [26]. This led to the proposition of the disinhibition model of signalling where the N-terminal domain inhibits constitutive activity through locking to the 7-TM domain, which upon activation, moves away from the receptor to increase signalling. This theory was challenged by the discovery of protease-activated receptor (PAR) activation mechanisms through the tethered agonist model [27]. PARs are cleaved by a number of endogenous proteases as well as proteases found in other species, resulting in a shorter N-terminal peptide [28] that can fold into the activation domain on the receptor. In 2014, two independent teams observed activation of aGPCRs by polypeptide fragments exposed post-cleavage, indicating that the tethered agonist model also applies to aGPCRs [29,30]. This further pointed to the conserved GAIN domain being responsible for autoproteolysis, contrary to PARs which require external proteolytic action. The GAIN domain helped explain the initial results of an increase in activity following cleavage as the cut sites were coincidentally located within the GAIN domain itself, mimicking the typical response of aGPCR activation [31].

**Figure 3.**
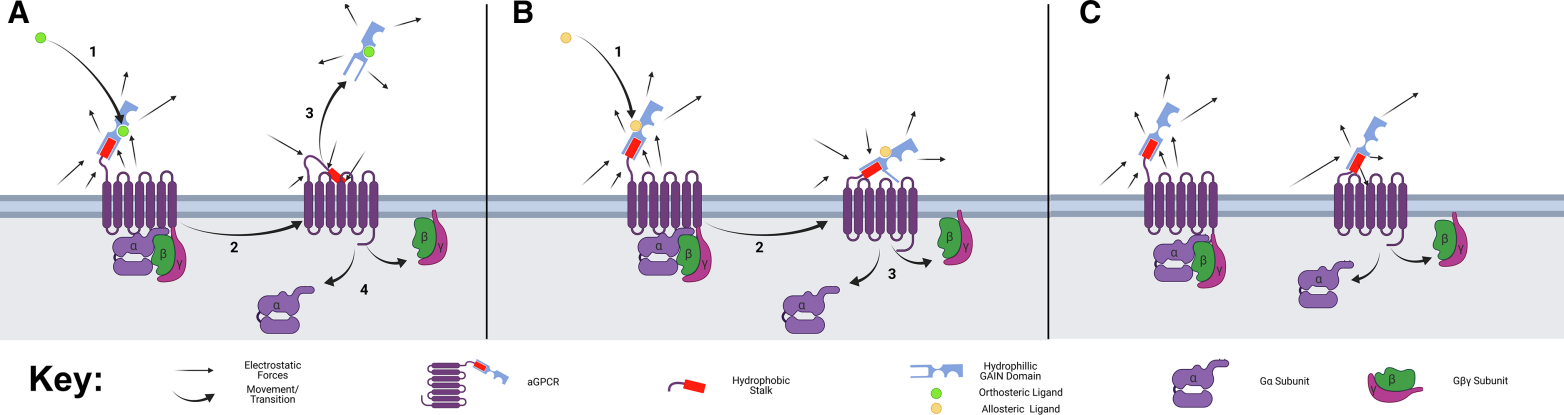
Proposed activation states of aGPCRs and the corresponding electrostatic forces. Inactive aGPCRs have their G proteins bound and stalks away from the activation domain in the centre of the GPCR. This is due to the hydrophilic GAIN domain still being attached and the hydrophobic stalk being hidden within it. (**A**) Full activation of the aGPCR is achieved by autoproteolysis of the GAIN domain, to expose the hydrophobic stalk to the ECM, pushing it toward the hydrophobic centre of the activation domain. This activates the GPCR releasing the G protein causing further downstream effects. (**B**) Partial allosteric activation can result in a conformational change of the GAIN domain resulting in the exposure of part of the hydrophobic stalk. This pushes the stalk toward the activation domain resulting in a higher chance of the G protein subunit dissociating. (**C**) Some receptors have constitutive activity, and this is likely due to the exposure of some of the hydrophobic residues on the stalk, resulting in more forces pushing the stalk away from the water rich ECM and toward the hydrophobic centre of the aGPCR. This can partially activate the aGPCR resulting in a higher chance of G protein subunit dissociation and downstream effects. Created using Biorender.

Although initial experimentation was difficult due to the hydrophobic nature of the fragments, cleavage and subsequent treatment using stalk fragments was successful. Single amino acid changes in the post cleavage stalk-peptides were discovered to have a variety of responses in aGPCRs, including inverse agonism [32]. This was partially explained with the stalk behaving like a lever and activity depending on its placement into the activation domain [33] (Figure 3A). This was given more credit thanks to the discovery of predicted β-turn elements within the stalk regions resulting in the stalk fragment bending into the receptor following cleavage [31]. The most recent theory suggests that the hydrophobic nature of the stalk contributes to the activity of the receptor, pushing it away from the aqueous ECD and into the relatively hydrophobic activation domain [22]. This does not explain all receptor activity however, with many noncleaved receptors still being able to signal in some capacity. Class B1 secretin-like GPCRs are also activated by soluble peptides such as glucagon and parathyroid hormone, exhibiting several activation states depending on the agonist present [34]. Therefore, it is likely that aGPCRs are also activated by allosteric agonists binding to the typically cleaved NTD. Currently, the exact mechanism is not known, but it may be postulated that agonist binding to an allosteric site could result in a conformational change of the NTD to push the stalk domain far enough into the activation domain of the receptor, eliciting activity through TMD to stabilse the active state (Figure 3B). Alternatively, there could be binding directly to the TMD stabilising it such as with cortisol and ADGRG3 (Table 1). This is further supported by the autoinhibitory nature of the GAIN domain with ligand binding to allosteric sites on the NTD relieving this action. Whilst orthosteric agonist activation is considered to result in full activation of the aGPCR, allosteric ligand binding can produce a graded response depending on the extent that the active site is stabilised. Many of these receptors have some degree of constitutive activity, without the need of an agonist present to elicit activity (Table 1 and Figure 3C). Finally, ligands may also bind to allosteric or orthosteric sites resulting in conformational changes that cause the opposite effect of typical agonism, also known as inverse agonism. These could move the stalk fragment away from the activation domain within the receptor, or destabilise the active site [35].

## N-terminal motifs of aGPCRs vary heavily and may determine ligand binding

The N-terminal regions of aGPCRs are consistently longer than those of Class A GPCRs, hence their initial grouping with Class B1. Their high Ser/Thr content, with many being glycosylated, gives the region a rigid, extended structure with high solubility, similar to mucin. Hence, one of the first identified aGPCRs was termed the EGF module-containing mucin-like hormone receptor (EMR1/ADGRE1) [36]. However, this feature is unlikely to directly influence ligand binding, as shown for GPR56 (ADGRG1) and one of its ligands, collagen III [37].

Many domains found in the aGPCR NTF are conserved features found in other proteins and across the aGPCR subfamilies (Table 1 — readers are directed to Hamann et al. [15] for a pictural representation of these NTFs). For instance, the most common feature is the hormone receptor domain (HomR), most commonly proximal to the GAIN domain [14]. This bears a striking sequence similarity to HomRs found in Class B GPCRs, to the extent that the latter may have descended from aGPCRs [38]. However, the GAIN domain has been shown to block the hormone-binding site of the ADGRL1 HomR [39], making their use for binding hormone ligands unlikely.

All cluster II aGPCRs contain EGF-like domains that vary only subtly between individuals. For example, the 3 amino acids that differ between ADGRE2 and 5 cause a huge bias of the ligand CD55 towards ADGRE5 [40]. Some EGF-like domains bind Ca^2+^, which is important for maintaining their structure and mediating protein ligand binding [41]. The Calx-beta motif, found in ADGRV1, also binds Ca^2+^ in the NTF, as demonstrated by their presence in Na^+^/Ca^2+^ exchangers [42]. From this, some have inferred that Calx-beta motifs could use Ca^2+^ to bind ligands, similarly to Ca^2+^-binding EGF domains [43]. Moreover, Complement C1r/C1s, Uegf, Bmp1 (CUB) domains have been demonstrated to use Ca^2+^ to bind ligands in various proteins [44,45], and have recently been shown to mediate intramolecular interactions in the ADGRG6 ECD. This gives a closed conformation by giving an interface between the CUB domain's tip and the more distal HomR that may contribute to the signalling state of the receptor [46].

aGPCR NTFs contain other domains and motifs found in a variety of proteins that are known to bind specific protein partners. Arginine-glycine-aspartate (RGD) motifs are known to bind integrins and are notably found in ADGRE5 [47–49]. Pentraxin (PTX) domains are found in a variety of aGPCRs, with variations between individuals that allow recruitment of specific ligands to specific receptors. For instance, the PTX and CUB domains of ADGRG6 have been shown to bind collagen IV, but not other collagen subtypes [50]. While not every identified NTF domain has been matched to a binding partner, the expansive repertoire of motifs and structures present demonstrate the heterogeneity of the ligands with which this family may interact.

Alternative splicing also expands this repertoire. The most variable part of aGPCR transcripts is the region N-terminal to the GAIN domain. Here, the position of individual domains can be altered, by addition of Ser/Thr stretches that vary NTF structure, or excluded entirely [51]. This is demonstrated in ADGRG6, where inclusion of 23 amino acids, many of which are glycosylated, disrupts this receptor's closed conformation, instead giving the receptor a more extended conformation that disrupts its ability to facilitate myelination *in vivo* [46].

## The GAIN domain separates aGPCRs into two fragments and may bind ligands

The GAIN domain is found almost ubiquitously in aGPCRs, between the variable N-terminal domains and the 7TM region, with only ADGRA1 lacking this region [14]. Its primary function is to allow receptor autoproteolysis at the GPS site, located proximally to the final β-strand (β13) of the GAIN_B_ subdomain (consensus: HLT, cleaving between L and T). These residues form a sharp turn, created by a disulfide bridge located proximally to the GPS and the Leu R-group being trapped in a hydrophobic pocket. Proteolysis is achieved by nucleophilic attack on the L-T peptide bond by the Thr R-group, with the resulting ester hydrolysing to give two separate fragments of the original protein [22,39,52].

There is also evidence for ligands binding to the GAIN domain to trigger aGPCR activation, such as CD90 binding ADGRE5 [49,53]. More recently, a small molecule agonist of ADGRF1, synaptamide, has been shown to interact with its GAIN domain [23]. These observations could explain the finding that cancer-causing mutations are found on the surface of the GAIN domain [39].

## The aGPCR 7TM domain retains recognised GPCR functions with novel motifs

Cryo-electron microscopy (cryo-EM) was recently used to elucidate the first full-length structure of an active aGPCR (ADGRG3) in complex with small molecule agonists (glucocorticoids cortisol and beclomethasone) and Gα_o_ [54]. The resultant structure had a 7TM region overall resembling that of a Class A GPCR, other than a greater separation between TM6 and TM7, giving a larger ligand-binding site. Extracellular Loop 2 (ECL2) forms a hydrophilic β-sheet that has a weak constitutive interaction with ECL3. This could act as a mechanism for relaying conformational changes from the NTF to the CTF upon ligand binding, allowing removal of the ECL2 ‘flexible lid’. This would expose the ligand binding pocket between TM6 and TM7, with the hydrophobic cores of glucocorticoid ligands packing against TM7. Alternatively, this feature may act to prevent dissociation of small molecule ligands by blocking their exit from the orthosteric site, slowing their dissociation rate and increasing the length of time over which the receptor signals, as seen in Class A GPCRs such as the endothelin-1 receptor B [55,56].

Unlike Class A GPCRs [57,58], ADGRG3 did not contain a core triad (IPF) motif or an NPxxY motif in TM7 that are involved in signal transduction across the GPCR. Instead of the core triad, ADGRG3 contained upper quaternary and lower triad cores (UQC and LTC) of hydrophobic residues that performed equivalent functions. A vital ‘toggle switch’ residue (W490^6.53^), contained within the UQC, recognises ligand binding and causes a conformational change that leads to its coupling to Gα_o_. Moreover, ADGRG3 lacks an ionic lock motif, normally found at the base of TM3 in Class A GPCRs (consensus: E/DRY), replacing it with a hydrophobic lock (HLY motif) that may perform similar roles in stabilising the receptor conformation on its cytoplasmic surface [54]. The differences in these key functional motifs between aGPCRs and other GPCR families further demonstrate the evolutionary distance between them, justifying the classification of aGPCRs as their own subfamily. The separation of the core triad into UQC and LTC between Family A and aGPCRs suggests a relatively distant common ancestor exists between the two. This structural difference also suggests the existence of novel methods for the design of small-molecule drugs targeting aGPCRs.

## aGPCRs activate a variety of effectors

GPCRs typically propagate their activation signal through two main classes of effectors: heterotrimeric G proteins, and β-arrestins resulting in an incredibly varied intracellular response profile [59]. ADGRF1 (GPR110), for example, is activated by synaptamide and can increase intracellular cAMP in a G_s_ dependent-manner as well as mobilise intracellular Ca^2+^ in a G_q/11_ dependent-manner, resulting in neurite growth and neurogenesis [60,61]. Interestingly, many of the downstream mediators and effectors of aGPCRs were discovered before their ligands, due to the self-cleavage aspect of their function. For example, ADGRG2 (GPR64) is currently an orphan receptor but due to manual cleavage of its NTD it has been observed to activate G_q_ mobilising intracellular Ca^2+^ [62] and G_s_ stimulating intracellular cAMP production [63]. ADGRG2 was also found to undergo β-arrestin-mediated endocytosis, further increasing its signalling repertoire by acting as a scaffold for downstream effectors.

β-arrestins are well known to mediate GPCR internalisation and activate numerous intracellular effectors for downstream signalling pathways, dependent on both the receptor itself as well as the ligand bound. Interestingly, β-arrestins can function in aGPCR signalling without full activation, which is atypical for many GPCRs [31]. The presence of the activating stalk in ADGRG1 was found to not be required for β-arrestin association, and therefore signalling. This could mean that allosteric agonism or even constitutive activity could be explained by arrestin recruitment and signal propagation. A further class of accessory protein recently discovered to interact with aGPCRs are the receptor activity-modifying proteins (RAMPs). RAMPs are a family of three single-pass transmembrane spanning proteins which were initially discovered to allow functional membrane expression and alter ligand specificity of the Class B1 GPCR calcitonin-like receptor (CALCRL) [64]. Since then, they have been discovered to interact with more GPCRs affecting receptor trafficking, downstream signalling and recycling [65,66]. Whilst the repertoire of RAMP-interacting GPCRs has expanded across Class A, B1 and C, in 2019, it was discovered that ADGRF5 (GPR116) interacts with RAMP2 and 3 [67]. Whilst the role of RAMPs in aGPCR function is currently unknown, this opens another avenue of research into aGPCR activity that may aide in the discovery of endogenous ligands which can only activate aGPCRs in the presence of RAMPs.

## aGPCRs have a multitude of physiological effects

As mentioned previously and reviewed extensively by Monk et al. [68], aGPCRs have significant function in paracrine signalling. They have a major role in the immune system, demonstrated by the large variety of aGPCRs found on immune cells [69]. These include ADGRB1 (BAI1) described above, which is required for the phagocytosis of apoptotic cells and pathogens in the brain. In addition to this, other aGPCRs such as ADGRG1 (GPR56) have been shown to be present in inflammatory natural killer cells along with cytotoxic lymphocytes [70]. Paracrine signalling is not limited to the immune system however, with several aGPCRs including ADGRL1 (Latrophilin-1) being suggested to increase synapse formation and function [71]. ADGRC1 (CELSR1) is another aGPCR responsible for dendritogenesis and axon guidance where KO studies have shown impaired migration of branchiomotor neurons during development [72]. One other area in which aGPCR function is also seemingly is required in the trafficking of stem cells to the bone marrow and their retention therein to produce haematopoietic cells, likely using soluble ligands due to the systemic trafficking of these cells [73].

## Clinical significance of aGPCR malfunction

aGPCRs have been implicated in numerous diseases, in particular, various types of cancers where a lack of function results in increased cell growth and metastasis [5]. One of the deadliest forms of cancer, lung cancer, can be severely affected by aGPCR mutations. ADGRB3 is an angiogenesis inhibitor, which has been found to be the most significantly mutated gene in 13% of lung squamous tumours, with mutations resulting in decreased activity of the receptor, increasing blood flow to the tumour [74]. Furthermore, it was discovered that in many lung cancers, the translation of ADGRB3 is decreased, resulting in reduced tumour suppressive effects provided by the receptor. Breast cancer was the second most diagnosed form of cancer in 2018 [75] and similarly to lung cancer, showed altered expression or mutation in aGPCRs. While typically not expressed in breast epithelial cells, ADGRE2 was shown to be up-regulated in invasive breast carcinomas and negatively correlated with survival and patient prognosis [76]. Previous research suggested ADGRE2 to have functions in the immune system therefore indicating further exploration is required in determining the secondary function in carcinoma progression. Recently, it was discovered that a further aGPCR, ADGRL4, promoted angiogenesis during both the development of the endothelium, as well as in several cancers where it is overexpressed. Of note is the lack of canonical GPCR signalling by this aGPCR, although several genes were found to have altered expression following activation suggesting an unusual method of signal transduction [24]. aGPCRs are quickly becoming a target of interest for other diseases outside of cancer, hopefully allowing for further harnessing of aGPCRs as therapeutic targets [73].

## Perspectives

- aGPCR research is a rapidly changing field with many orphan aGPCRs and an emerging picture of how agonists cause receptor activation. Further insights into these may help in rational drug design for aGPCRs. This would aid in treatments of diseases which aGPCRs are involved in, such as cancer, due to their control over angiogenesis and up-regulation in breast cancer.
- The current consensus on aGPCR activation involves the GAIN domain acting in an autoinhibitory way to occlude a tethered ligand, as demonstrated by constitutively active aGPCR mutants. Ligands may bind to the NTF to cause the tethered ligand to be exposed, allowing its binding to the aGPCR orthosteric site and receptor activation. Recent cryo-EM studies have shown that, from here, aGPCR conformational changes reflect those in Class A GPCRs, but make use of different motifs.
- Future directions may include the cryo-EM analysis of more aGPCRs to allow comparative structural studies demonstrating the importance of sites such as the UQC; further elucidation of how the binding of ligands to the NTF can cause receptor activation; and using this newfound knowledge of aGPCR activation to design drugs to alter their activity.

## Open Access Statement

Open access for this article was enabled by the participation of University of Cambridge in an all-inclusive Read & Publish pilot with Portland Press and the Biochemical Society under a transformative agreement with JISC.

## Abbreviations

CUB: C1r/C1s, Uegf, Bmp1
ECD: extracellular domain
ECL2: extracellular loop 2
EGF: epidermal growth factor
GAIN: GPCR autoproteolysis-inducing
GPS: GPCR proteolysis site
LPA1: lysophosphatidic acid receptor
PS: phosphatidylserine
PTX: Pentraxin
RAMPs: receptor activity-modifying proteins
TM: transmembrane
